# Development and Validation of the Gay-Specific Intraminority Stigma Inventory (G-SISI): Initial Evidence Underpinned by Intraminority Stress Theory

**DOI:** 10.3390/ejihpe13010013

**Published:** 2023-01-12

**Authors:** Benjamin F. Shepherd, Justin L. Maki, David G. Zelaya, Şeniz Warner, Adriana Wilson, Paula M. Brochu

**Affiliations:** 1 Department of Clinical and School Psychology, Nova Southeastern University, Fort Lauderdale, FL 33314, USA; 2 School of Public Health, Brown University, Providence, RI 02903, USA; 3 Department of Psychiatry, Harvard Medical School, Boston, MA 02215, USA

**Keywords:** intraminority stigma, intraminority gay community stress theory, intersectionality, community involvement, gay men

## Abstract

There is currently a lack of measures testing intraminority stress within gay men. Therefore, the current study sought to develop and psychometrically test the Gay-Specific Intraminority Stigma Inventory (G-SISI). Based on a content review of the literature and a panel of experts, a pool of items assessing gay men’s perceived exposure to a range of discriminatory attitudes from other gay men was generated. Utilizing a randomly split sample of 1723 gay men between the ages of 19 and 79 years, an exploratory factor analysis was first performed (*n* = 861). The remaining unexamined data were then used to conduct a confirmatory factor analysis (*n* = 862). The results support a six-factor model: (1) Age Stigma, (2) Socioeconomic Stigma, (3) Gay Non-Conformity Stigma, (4) Racial Stigma, (5) Gender Expression Stigma, and (6) Body Stigma. Cronbach’s alpha for the total scale was 0.90 and for the subscales ranged from 0.60 to 0.85. Sociodemographic factors and measures of community involvement were differentially associated with the G-SISI subscales, providing evidence of construct validity. The findings demonstrate initial support for the dimensionality and validity of the G-SISI, which targets modifiable factors (e.g., identity-based stigma) that may increase stress and reduce community coping resources among gay men with diverse identities.

## 1. Introduction

### 1.1. Community Resilience and Risk

It is well established that human beings share a fundamental need for social safety, defined as reliable social connection, inclusion, and protection, all of which are threatened by stigma [1,2,3,4]. Stigma refers to the negative or discriminatory attitudes aimed at an individual or group based on an identity or characteristic that is socially marginalized [5]. Across the lifespan, stigma can assume many forms, including family rejection [6,7], peer bullying [8,9], and non-inclusive healthcare [10,11,12]. Such identity-based threats have the potential to adversely impact an individual’s self-concept [13,14], psychosocial functioning [15,16], and health [4,17,18,19]. However, several scholars assert that deriving strength and solidarity through identity-based group memberships can help prevent stigma and other life stressors from “getting under the skin” [19,20,21,22,23].

Among gay men, the gay community can serve as an important source of belonging, support, and resilience in a variety of ways [20,24,25,26,27]. For example, queer establishments and gatherings (e.g., community activities, nightlife) can provide physical spaces for gay men to safely reveal their sexual identity and connect with each other both socially and sexually, offering a temporary escape from the heteronormativity of society [24,25,28]. Similarly, online networking platforms geared toward gay men (e.g., dating apps) also provide opportunities for coping, granting users of these services identifiable and anonymous access to their community, which may be particularly useful for men who fear exploring or disclosing their sexual identity publicly [25,29,30].

Although forming strong ties to the gay community is traditionally conceptualized as a protective factor that bolsters self-esteem and buffers against the stress of discrimination, anxious expectations of rejection, and other minority stressors (e.g., sexual identity concealment, internalized homonegativity) [19,20,24,25], a growing body of studies with diverse methodologies indicates that “gay spaces” do not always provide the same level of social safety to all gay men (e.g., gay men of color, higher-weight gay men) and may be a separate source of stress [30,31,32,33,34,35]. For example, Convertino and colleagues [36] found that, in addition to traditional minority stressors, community involvement (i.e., the degree to which a person participates in the LGBTQIA+ community) is associated with body image-related concerns and behaviors such as disordered eating and appearance- and performance-enhancing drug misuse. One theoretical perspective that may elucidate these findings is intraminority gay community stress theory.

### 1.2. Intraminority Gay Community Stress

Intraminority gay community stress theory [35] posits that men with minoritized sexual identities (e.g., gay men) experience unique status-based, competitive pressures flowing from a reliance on other men to meet their social and sexual needs. The theory was recently developed and tested by Pachankis and colleagues [35], who interviewed a diverse group of gay and bisexual men and developed a multifactorial measure of intraminority gay community stress (i.e., the Gay Community Stress Scale). The scale examines perceived stress related to the gay community’s focus on sex (e.g., valuing sex over meaningful relationships), status (e.g., overly valuing men who are wealthy), competition (e.g., having a culture of competition and jealousy), and exclusion of diversity (e.g., being racist). As hypothesized, these stressors were found to vary by status-relevant sociodemographic factors (e.g., income, masculinity, race/ethnicity), thwart feelings of belonging, and confer risk for mental health concerns over and above the effects of traditional minority stressors (e.g., heterosexist stigma).

The Gay Community Stress Scale in its full [35] or abbreviated [37] form is a valuable measure that robustly captures gay men’s current level of stress in relation to their perceptions about various aspects of the mainstream gay community (i.e., popular gay culture). Since these concerns are rooted in stigmatizing social structures and stereotypes, their salience is likely influenced by lifetime experiences of direct exposure to social disadvantage and marginalization from within the community [33,34]. However, the scale is not designed to explicitly measure the frequency with which gay men experience discriminatory attitudes from other gay men in their daily lives, which may shape perceptions of the mainstream gay community and related stress [33,38]. Additional research is critical to target the modifiable factors (i.e., identity-based stigma) that contribute to sociodemographic imbalances in intraminority stress and, in turn, an inequitable distribution of community coping resources (e.g., belonging, support) [34,35,39]. These resources, when obtained, are shown to be particularly important among multiply marginalized gay men [40].

### 1.3. Intraminority Stigma

Intraminority stigma occurs when, instead of promoting solidarity, less privileged members of a marginalized group (e.g., the gay community) are othered or devalued by ingroup members [23,41]. For example, as a product of systemic racism, gay men of color tend to perceive gay spaces as mainly “White spaces,” face recurring discrimination on dating apps and websites, and may avoid or have trouble forming connections with other gay men [42,43]. In line with intraminority stress theory, racial stigma experienced within the supposed safety of non-heteronormative contexts is consistently linked to elevated stress and other mental health-related concerns among individuals with minoritized sexual and racial identities [31,39,42,44,45].

Intersectionality, a critical analysis originally developed by Crenshaw [46,47], provides a lens to examine how multiple systems of oppression collectively interact to create disparities in sociocultural power and privilege among individuals who live at the nexus of possessing multiple diverse identities. Such interactions result in unique obstacles and experiences (e.g., intraminority stigma) that can be quantified to advance social and psychological science [48]. Yet, there are no current measures that concomitantly examine multiple constructs of intraminority stigma from the perspective of gay men. A multifaceted instrument designed to gather information on a wide range of intersectional challenges encountered within the context of the gay community allows for a more comprehensive assessment of the unique concerns and needs of gay men with diverse identities, and has important research, clinical, and policy implications for ameliorating social and psychological health inequities within this population.

### 1.4. Current Study

An extensive review of the literature identified six constructs of intraminority stigma that appear to be prevalent within the gay male community [49]. Based on this review and a panel of seven LGBTQ+ experts with a doctorate in counselor education, a new self-report measure was developed to quantify the frequency with which gay men experience an array of discriminatory attitudes from other gay men using an intersectional approach. Although there is a previous scale (i.e., the Gay Community Stress Scale) that examines intraminority stress within gay and bisexual men [35], the scale assesses for perceived level of stress related to perceptions of broad themes of rejection and exclusion, whereas the novel measure created here, titled the Gay-Specific Intraminority Stigma Inventory (G-SISI), examines intraminority stress based on the perceived level of exposure to specific forms of identity-based stigma.

To provide psychometric support for the G-SISI, the following hypotheses were formulated and tested. First, it was hypothesized that an exploratory factor analysis will reveal six subscales representing six clearly delineated constructs of intraminority stigma (Hypothesis 1), a confirmatory factor analysis will confirm the factor structure (Hypothesis 2), and adequate internal consistency reliability will be demonstrated (Hypothesis 3).

Next, to test construct validity, we posited that gay men’s scores on the G-SISI will differ by sociodemographic characteristics. Based on the reviewed literature, it was hypothesized that intraminority age stigma will be most commonly experienced by older gay men relative to younger gay men (Hypothesis 4) [50,51]; intraminority socioeconomic stigma will be most commonly experienced by gay men with lower socioeconomic status relative to gay men with higher socioeconomic status (Hypothesis 5) [52,53]; intraminority gay non-conformity stigma will be most commonly experienced by politically conservative gay men (e.g., Republican) relative to politically liberal gay men (e.g., Democrat; Hypothesis 6) [54]; intraminority racial stigma will be most commonly experienced by gay men of color relative to White gay men (Hypothesis 7) [31,43]; intraminority gender expression stigma will be most commonly experienced by gay men who express themselves more femininely relative to gay men who express themselves more masculinely (Hypothesis 8) [55,56]; and intraminority body stigma will be most commonly experienced by gay men with a higher weight relative to gay men with a lower weight (Hypothesis 9) [33,57].

Lastly, we posited that gay men’s experiences of intraminority stigma will differ by frequency of attendance at gay-specific establishments/gatherings and dating app/website usage, as prior research theorizes that gay men who are more embedded within gay spaces, especially for romantic or sexual reasons, experience higher levels of intraminority stress (Hypothesis 10) [35,58].

## 2. Materials and Methods

### 2.1. Participants

Participants who self-identified as gay men and had internet access were eligible to participate in the study. The minimum age to participate was 19 years to account for states where 19 was the age of majority for participation in research (e.g., Alabama). Those who did not identify as a gay man, were under the age of 19, did not complete the study in its entirety, or resided outside of the United States (*n* = 463) were excluded from this sample. A total of 1723 participants were randomly split into two separate samples to conduct an exploratory factor analysis (EFA) and a confirmatory factor analysis (CFA). Participants ranged in age from 19 to 79 years in the EFA sample (*M* = 41.87, *SD* = 13.36) and from 19 to 74 years in the CFA sample (*M* = 41.35, *SD* = 13.36). Across both samples, a majority of participants identified as White, Democrat, middle-class, more masculine than feminine, above average weight, and single. For more information regarding the sociodemographic characteristics of the sample, see Table 1.

### 2.2. Instrument

A pool of 24 items assessing various experiences of gay-specific intraminority stigma was generated based on a content review of the literature [49]. Following best practice recommendations by Boateng and colleagues [59], the content validity of these items was evaluated and approved by a panel of experts (i.e., seven LGBTQ+ counselor educators) before being administered to participants. Participants were asked to “respond to statements based on their experience/s with another gay man or group of gay men,” such as “I have been told I need to behave more masculine or feminine” and “I have been told I should gain or lose weight,” using a 5-point scale ranging from 1 (never) to 5 (very frequently). Higher scores indicate more frequent experiences of intraminority stigma. The items were designed to represent six constructs of stigma that gay men may experience and perpetuate (i.e., age stigma, socioeconomic stigma, gay non-conformity stigma, racial stigma, gender expression stigma, and body stigma).

### 2.3. Demographic Questionnaire

Participants were asked to provide information about their age, socioeconomic status, political affiliation, race/ethnicity, gender expression, and perceived weight. Socioeconomic status was measured on a 5-point scale ranging from 1 (lower class) to 5 (upper class). Gender expression was measured on a 5-point scale ranging from 1 (very feminine) to 5 (very masculine). Perceived weight was measured on a 5-point scale ranging from 1 (underweight) to 5 (overweight). For race/ethnicity, the response options included “African American/Black,” “Hispanic/Latino,” “White,” “Asian,” “American Indian,” “Hawaiian/Pacific Islander,” and “Multiracial/biracial.” Participants also had the option of specifying another race/ethnicity. For political affiliation, the response options included “Republican,” “Democrat,” “Libertarian,” and “Independent.” Participants also had the option of specifying another political affiliation. For relationship status, the response options included “single,” “in a relationship,” “married,” “divorced,” and “widowed.” Due to samples size restrictions, race/ethnicity (i.e., Person of Color or White) was analyzed as a dichotomous variable, and political affiliation (i.e., Republican, Democrat, Independent) was analyzed as a trichotomous variable.

In addition, the demographic questionnaire consisted of two items that assessed different types of community involvement (in person and online) that were developed for this study, consistent with prior research [60]. Participants’ frequency of attendance at gay-specific establishments and/or gatherings was assessed on an 8-point scale ranging from 1 (never) to 8 (daily). Higher scores indicate more frequent attendance at gay-specific establishments and/or gatherings. Similarly, a single item was used to assess the frequency with which participants used dating apps/websites to meet and/or hook up with other gay men (i.e., “How often do you visit gay dating and/or hook-up apps/sites?). Possible responses included: “never” (1), “less than once a year” (2), “every six months” (3), “every 2–3 months” (4), “monthly” (5), “weekly” (6), “more than a few times a week” (7), and “daily” (8). Higher scores indicate more frequent dating app/website usage.

### 2.4. Procedure

In a series of steps, gay- and male-identifying individuals were recruited via social media advertisements (e.g., Facebook pages geared towards gay men) to participate in an anonymous Qualtrics survey containing the aforementioned instrument and demographic questionnaire. First, a search on Facebook was conducted to identify groups that were only open to gay men. Once the groups were identified, the group administrators were contacted to seek permission for posting a link to the online survey. Additionally, support groups for the LGBTQ+ community were contacted for permission to post the link to the survey. The survey was available over a four-week period; prior to beginning the survey, respondents were asked to provide their informed consent. Data collection was completed in 2018 as a part of a larger study [49]. All procedures were approved by the Institutional Review Board at Auburn University.

### 2.5. Data Analysis Plan

Using SPSS (v. 27, SPSS Inc., Chicago, IL, USA), data were first cleaned to satisfy exclusion criteria, then statistically checked for missing values and normality before running further analyses. Across the variables of interest, no missing values were found, and there is evidence that the normality assumption was met, given all skewness and kurtosis values fell within an acceptable range (between −2 and +2) [61]. For sample sizes larger than 300, this method of determining substantial non-normality is recommended [62].

Next, the total sample was randomly split into two separate files to conduct the EFA (*n* = 861) and the CFA (*n* = 862). For the EFA, the Kaiser–Meyer–Olin index (KMO) and Barlett’s test of sphericity were used to ensure data were appropriate for factor analysis. Factor loadings and eigenvalues were obtained using principal axis factoring. Factor extraction was based on the Kaiser criterion, which states that factors with eigenvalues greater than or equal to one should be retained, and the scree plot examined. The pattern matrix was examined for conceptual and psychometric support in the deletion and retention of items following recommendations by Worthington and Whittaker [63]. Factor loadings were examined for interpretability based on low loadings, defined as 0.40 or less, and cross-loadings, defined as a second factor loading of 0.30 or greater. Weak factor loadings (i.e., less than 0.40) indicate that a particular item may be less relevant in measuring a particular construct.

Sample size recommendations suggest a minimum of 300 participants for complex models [64]. Therefore, our sample size of 862 is adequate for CFA testing. Model fit was evaluated using the following guidelines: a non-significant chi-square, root-mean-square error of approximation (RMSEA; less than 0.10), standardized root-mean-square residual (SRMR; less than 0.10), and comparative fit index (CFI; greater than 0.90) [65]. The covariance matrices were examined using maximum likelihood estimation and the standardized solutions were interpreted using Mplus Version 8.2 software [66].

To establish construct validity, participants’ scores on the G-SISI were examined across sociodemographic characteristics (i.e., age, socioeconomic status, political affiliation, race/ethnicity, gender expression, perceived weight) and embeddedness within gay men’s social and sexual spaces (i.e., frequency of attendance at gay-specific establishments and dating app use) using bivariate correlations, independent sample *t*-tests, and an analysis of variance (ANOVA). For the ANOVA, when significant main effects were detected, Tukey-corrected post hoc comparisons were performed to determine which groups differed from each other. For the independent sample *t*-tests, the effect size was analyzed with Cohen’s *d*. Based on criteria suggested by Cohen [67], effects were interpreted as small when *d* = 0.20, medium when *d* = 0.50, and large when *d* = 0.80. For the bivariate correlations, the effects were interpreted as small when *r* = 0.10, medium when *r* = 0.20, and large when *r* = 0.30 [68].

## 3. Results

### 3.1. Exploratory Factor Analysis (Hypothesis 1)

A total of 24 items were entered into an EFA, which was conducted with a Promax rotation to determine the underlying factor structure of the G-SISI. The Keiser–Meyer–Olin (KMO) value of sampling adequacy fell within the acceptable range (0.92), above the recommended criterion of 0.50 [69], and the Bartlett test of sphericity was statistically significant, χ^2^(276) = 8499.37, *p* < 0.001, indicating the data are factorable [70]. The eigenvalue for six factors ranged from 7.904 to 1.02, exceeding the recommended criterion of 1. The seventh factor did not (0.88). The scree plot was examined and exhibited an elbow between the sixth and seventh factor, suggesting a six-factor solution. Subsequently, four items were dropped due to low factor loadings or cross-loadings onto another factor. The final EFA resulted in 20 items being retained, comprising six factors which were called the: Age Stigma subscale (factor 1), Socioeconomic Stigma subscale (factor 2), Gay Non-Conformity Stigma subscale (factor 3), Racial Stigma subscale (factor 4), Gender Expression Stigma subscale (factor 5), and Body Stigma subscale (factor 6), accounting for 32.92%, 9.30%, 6.74%, 4.87%, 4.50%, and 4.25% of the variance, respectively.

### 3.2. Confirmatory Factor Analysis (Hypothesis 2)

The six-factor model yielded a good fit to the data: χ^2^ (155) = 424.47, *p* < 0.01; RMSEA = 0.05 (95% CI: 0.04–0.05), SRMR = 0.04, CFI = 0.95. We moved forward with interpreting the model regardless of the significant chi-squared test, as researchers have previously noted that the chi-squared test is sensitive to sample size [71]. All items significantly loaded onto their latent factor (*p* < 0.001; see Table 2). Latent variable correlations between the six factors ranged from 0.39 to 0.74.

### 3.3. Internal Consistency (Hypothesis 3)

The Cronbach’s alpha for the overall measure was 0.90 and Cronbach’s alpha for the six factors was mostly within the acceptable ranges [72]: Age Stigma subscale (α = 0.80), Socioeconomic Stigma subscale (α = 0.78), Gay Non-Conformity Stigma subscale (α = 0.60), Racial Stigma subscale (α = 0.81), Gender Expression Stigma subscale (α = 0.85), and Body Stigma factor subscale (α = 0.77).

### 3.4. Construct Validity (Hypotheses 4–10)

The scores of the total scale and subscales of the G-SISI were examined in relation to several sociodemographic characteristics and community involvement variables (i.e., attendance at gay-specific establishments or gatherings and use of gay-specific dating apps or websites) to establish convergent validity. Of note, all six subscales measuring different forms of intraminority stigma were significantly correlated with each other in the positive direction (all *p*s < 0.001); the magnitude of these correlations were large (all *r*s > 0.30; see Table 3).

#### 3.4.1. Sociodemographic Characteristics

As hypothesized, participant age was positively correlated with the Age Stigma subscale: *r*(860) = 0.26, *p* < 0.001. Participant socioeconomic status was positively correlated with the Socioeconomic Stigma subscale: *r*(860) = −0.26, *p* < 0.001. There was a significant main effect of participant political affiliation on the Gay Non-Conformity Stigma subscale: *F*(2859) = 28.24, *p* < 0.001, η_p_^2^ = 0.06. Specifically, Republican gay men (*M* = 3.26, *SD* = 1.08) reported significantly greater experiences of gay non-conformity stigma from other gay men than Democrat gay men (*M* = 2.53, *SD* = 0.87; *t*(588) = 5.64, *p* < 0.001, Cohen’s *d* = 0.81) and politically independent gay men (*M* = 2.92, *SD* = 0.89; *t*(323) = 2.42, *p* = 0.016, Cohen’s *d* = 0.36). Politically independent gay men also reported higher scores on the Gay Non-Conformity Stigma subscale than Democrat gay men: *t*(807) = 5.96, *p* < 0.001, Cohen’s *d* = 0.44. Participants’ scores on the Racial Stigma subscale differed by participant race/ethnicity (*t*(860) = 18.21, *p* < 0.001, Cohen’s *d* = 1.39), such that gay men of color (*M* = 2.93, *SD* = 1.07) experienced more intraminority racial stigma than White gay men (*M* = 1.79, *SD* = 0.70). Participant masculinity was negatively correlated with the Gender Expression Stigma subscale: *r*(860) = −0.40, *p* < 0.001. Participant perceived weight was positively correlated with the Body Stigma subscale: *r*(860) = 0.28, *p* < 0.001.

#### 3.4.2. Attendance at Gay-Specific Establishments or Gatherings

Frequency of attendance at gay-specific establishments or gatherings was positively correlated with the Racial Stigma subscale (*r*(860) = 0.09, *p* = 0.012) but not significantly correlated with any other measure of intraminority stigma: all *r*s(860) < |−0.06|, *p*s > 0.117.

#### 3.4.3. Use of Gay-Specific Dating Apps or Websites

As expected, the use of gay-specific dating apps or websites was positively correlated with cumulative reports of gay-specific intraminority stigma experiences: *r*(860) = 0.09, *p* = 0.007. Specifically, dating app/website usage was positively correlated with the Age Stigma subscale (*r*(860) = 0.08, *p* = 0.027), the Socioeconomic Stigma subscale (*r*(860) = 0.07, *p* = 0.030), the Racial Stigma subscale (*r*(860) = 0.10, *p* = 0.005), and the Body Stigma subscale (*r*(860) = 0.11, *p* < 0.001). By contrast, dating app/website usage was not significantly correlated with the Gay Non-Conformity Stigma subscale (*r*(860) = 0.02, *p* = 0.625) or the Gender Expression Stigma subscale (*r*(860) = 0.03, *p* = 0.471).

## 4. Discussion

The G-SISI was developed to assess the dimensionality and frequency of gay men’s experiences of intraminority stigma. Overall, the results from the factor analyses, internal consistency estimates, and tests of construct validity support the use of this six-factor, 20-item measure with gay men. Accounting for more than 60% of the total variance in the factor analyses, the G-SISI reflects a range of social messages and pressures potentially perceived by gay men with diverse identities, including stereotypical comments, criticisms, and acts of discrimination based on one’s age (factor 1), socioeconomic status (factor 2), interests, hobbies, or beliefs (factor 3), race/ethnicity (factor 4), gender expression (factor 5), and body size and shape (factor 6). These facets are consistent with and expand upon themes in the theoretical and empirical literature discussing experiences of gay-specific intraminority concerns (e.g., youth-driven, socioeconomic status, career success, judgment/criticism, racial stereotypes, idealizing hyper-masculinity, body image) [35].

### 4.1. Sociodemographic Differences in Intraminority Stigma Experiences

As expected, gay-specific intraminority stigma experiences were differentially associated with sociodemographic characteristics in our sample. Older age was associated with more frequent experiences of intraminority age stigma. This complements prior research on ageism within the gay community, underscoring the need to increase access to community coping resources for men of all ages [50]. Intraminority age stigma may affect mental health via internalized gay ageism (i.e., the sense that one feels denigrated or depreciated because of aging in the context of a gay male identity) [51].

Socioeconomic status, political affiliation, and race/ethnicity were associated with the Socioeconomic Stigma subscale, Gay Non-Conformity Stigma subscale, and Racial Stigma subscale, respectively, such that gay men with lower SES reported greater experiences of intraminority socioeconomic stigma (relative to gay men with higher SES), Republican gay men reported greater experiences of intraminority gay non-conformity stigma (relative to Democrat and politically independent gay men), and gay men of color reported greater experiences of intraminority racial stigma (relative to White gay men). These findings were expected considering that the mainstream gay community is predominantly comprised of and places a higher value on middle-class, liberal-minded, and White individuals [35,42,52,54].

Similarly, consistent with prior research on gay men’s experiences of femmephobia [55,56] and anti-fat bias [33,73] from potential romantic or sexual partners, masculinity and weight were associated with experiences of intraminority stigma. Specifically, gay men who identified as more masculine than feminine reported fewer experiences of gender expression stigma from other gay men relative to their more feminine counterparts, and gay men who perceived themselves as being higher-weight reported more experiences of body stigma from other gay men relative to their lower-weight counterparts. Taken together, these findings suggest gay men with diverse identities are at increased risk for experiencing rejection and exclusion from other gay men as a product of stigmatizing social structures and stereotypes that pervade society and, by extension, the gay community.

### 4.2. Intraminority Stigma and Community Involvement

Consistent with prior scholarly work showing that gay dating app users may express their sexual “preferences” directly on their profiles, systematically ignore messages, or explicitly reject other users based on a number of status-relevant attributes such as race [42] and weight [73], the greater use of dating/hookup apps or websites was associated with more frequent experiences of intraminority stigma, particularly racial stigma, body stigma, age stigma, and socioeconomic stigma.

Interestingly, more frequent attendance at gay-specific establishments or gatherings was associated with intraminority racial stigma, but no other form of intraminority stigma, highlighting the well-documented lack of racial representation and inclusion within these spaces [31,43]. In general, gay men have control over what establishments and gatherings they choose to attend or avoid, some of which may be more welcoming of diversity than others, and are often accompanied by friends, acquaintances, or partners, whereas gay dating apps are more anonymous and less predictable, designed specifically to connect gay men with potential romantic or sexual partners rather than foster a sense of community belonging [30,58]. In fact, some research shows that more frequent use of gay dating apps (e.g., Grindr) is associated with a lower sense of community, higher levels of loneliness, and lower levels of life satisfaction, as well as body image-related issues—possibly as a function of intraminority stigma [30,73]. Moreover, Pachankis and colleagues [35] found that intraminority stress disproportionately affects single gay men relative to their partnered counterparts, further suggesting intraminority stigma may be more prevalent within the context of gay-specific dating/hookup apps and websites.

Overall, the current findings provide quantitative and contextual evidence of how “gay spaces”—though intended for gay men to safely socialize—may not actually be safe for all gay men and may perpetuate hierarchies of power and stigma that extend beyond the gay community. Thus, it is important to consider the valence of social interactions between gay men in addition to frequency, as well as the influence of intersectionality, when assessing psychosocial risks within this population [42,45].

### 4.3. Intersectional Considerations

Consistent with intersectionality theory [46,47,48], all six factors were positively associated with each other, united by interlocking systems of oppression that distribute power and privilege unevenly across sociodemographic characteristics. As such, the sums of different dimensions of identity and stigma are not necessarily equal to or greater than the intersectional experience and thus should be interpreted with caution [74]. This may explain why associations between experiences of intraminority stigma and different forms of community involvement were either small or non-significant in our sample. It is likely that higher levels of community involvement—whether in person or online—would pose a greater risk to subgroups of gay men with marginalized social positionality rather than the full sample. For example, it is well documented that gay men who express themselves more femininely are discriminated against much more frequently on mobile dating sites by potential partners than their more masculine counterparts (e.g., writing “masculine guys only” directly in their profiles) [56]; however, a majority of the participants in our sample identified as more masculine than feminine. Likewise, most of the sample was White, resulting in weaker associations between intraminority racial stigma and community involvement. More intersectional research is needed to examine how multiple facets of identity, stigma, and community involvement may interact to differentially predict the health and well-being of gay men with diverse identities [25,42].

### 4.4. Limitations and Future Directions

The results of the current study must be considered within the context of limitations of the design and sample. First, the G-SISI requires further validation (e.g., measurement invariance, psychometric testing with more diverse samples) before drawing firm conclusions [74]. Recent studies examining the potential consequences of intraminority stress suggest experiences of gay-specific intraminority stigma may be associated with depression, anxiety, and somatic symptoms [35], social anxiety [75], and sexual risk taking [76]. As such, the convergent validity of the G-SISI could be bolstered by future research testing the associations of the measure with various health outcomes (e.g., mental health, physical health), as well as with measures of intraminority gay community stress [35,37], community inequity [34], and additional status-relevant sociodemographic characteristics (e.g., income, level of muscularity).

Concurrent validity could be tested at the subscale level. For example, the LGBT People of Color Microaggression Scale [77,78,79] can be compared to the Intraminority Racial Stigma subscale. In addition, the G-SISI could be extended to include more items and forms of intraminority stigma, such as intraminority stigma based on HIV status [80], disability status [81], and consensual non-monogamy [82]. Ponterotto and Ruckdeschel [83] note that scales with few items may have difficulty reaching a high magnitude of internal reliability, as was evident within the Gay Non-Conformity Stigma subscale that only has three items.

Generalizations cannot be made regarding the pervasiveness of intraminority stigma among gay men living in the United States or other countries because a nationally representative sample was not surveyed. With respect to the gender diversity of the sample, all participants were self-identified gay men, and thus some participants may have been transmasculine. Consequently, this study’s assessment of gender expression may have captured differences in gender expressiveness between cisgender and transgender gay men. Future studies are encouraged to use a two-step method (i.e., gender identity, assigned sex at birth) or other gender-inclusive data-collecting strategies to better understand how the prevalence and impact of gay-specific intraminority stigma may differ among gender-diverse/expansive populations [84,85].

The lack of racial/ethnic diversity in the sample further limits the generalizability of the findings, as well as the power to detect within-group differences between minoritized racial/ethnic groups. Future studies should recruit more racially/ethnically diverse participants and consider participants’ level of connectedness to their respective racial/ethnic communities, including queer communities of color, as a possible source of community resilience and coping against gay-specific intraminority stigma [39,42,86].

Similarly, it might prove fruitful to gather information regarding participants’ identification and involvement with gay subcultures (e.g., the Bear community) [35,87,88,89], which present opportunities for identity-based group memberships that endorse different standards that are not yet valued by the mainstream gay community and may partly explain why attendance at gay-specific establishments or gatherings was not predictive of every form of intraminority stigma. Alternatively, belonging to some gay subcultures may be a source of shame, rejection, and distress for some gay men as a result of status-based hierarchies [35,87,88].

Given previous research indicating greater involvement with the gay community may be a risk factor for various health-risk behaviors, such as increased body surveillance and substance use [25,30,36,73], it is recommended that gay-specific intraminority stigma be tested as a mediator to elucidate these associations [42]. Furthermore, some evidence suggests exposure to a greater number of forms of stigma is associated with greater mental distress and a lower level of well-being among individuals with multiple marginalized identities [90,91]. Future research could test which constellations of constructs of the G-SISI are more strongly associated with which health outcomes [92]. For example, intraminority body stigma may become internalized and drive extant eating disorder disparities faced by gay men [93,94], or perhaps the association between intraminority stigma and health is more transdiagnostic in nature [95].

### 4.5. Implications for Practice and Policy

In addition to paving the way for future research, the G-SISI has important clinical and policy implications. Gay men are at increased risk of experiencing mental health concerns, risky substance use, disordered eating, and self-injurious behaviors [18,94,95,96,97,98]. Further, Dziengel [99] reported that the number of gay men and lesbian women who seek counseling services related to their sexual minority status could be as high as 42% of that population, yet this population is twice as likely to report dissatisfaction with a mental health service compared to their heterosexual peers [100]. With the elevated utilization of and dissatisfaction with mental health services among non-heterosexual persons, it is essential to understand more about their lived experiences [101]. Gathering information about multiple aspects of identity and experiences of intraminority stigma—in addition to heterosexist stigma—can help clinicians gain more insight into the intimate lives of their clients who identify as gay men, as well as their presenting concerns, and how to tailor individual-level interventions accordingly [102,103,104]. For example, a recent qualitative study of Black men who have sex with men identified pride in intersectional identities, perseverance, community advocacy, and social support as adaptive responses to heterosexist and racist stigma [105], all of which can be fostered in therapy.

Considering community-level and systems-level approaches to tackling the issue of intraminority stigma as a modifiable point of intervention, such as the development of programs and policies aimed at creating more welcoming, affirmative, and equitable environments and promoting intraminority solidarity, is also critical to support adaptive coping and resilience among gay men with diverse identities [23,106,107,108,109]. In fact, in a seven-year, multinational study, life satisfaction improved over time as a function of reductions in country-level structural stigma among gay men and other sexually diverse individuals, especially those who were in a relationship [110]. With a comprehensive understanding of stigma perpetuated both outside and within the gay community, researchers, practitioners, policymakers, community advocates, and other agents of change are better equipped to address the unique, intersectional concerns within this population.

## 5. Conclusions

We have developed and provided initial support for the multidimensionality and validity of a self-report measure that assesses direct exposure to multiple forms of stigma that gay men may experience concomitantly from within the gay community. Underpinned by intraminority gay community stress theory [35], the G-SISI has the potential to detect risks related to social disconnection and stress, as well as intersectional resilience. Future research is encouraged to use the G-SISI as a culturally responsive roadmap that considers multiple aspects of identity and interlocking systems of oppression to uncover better ways of meeting the psychosocial needs of multiply marginalized gay men.

## Figures and Tables

**Table 1:** Sociodemographic Characteristics.

Variable	EFA Sample (*n* = 861)		CFA Sample (*n* = 862)	
	%	*N*	%	*n*
Race/Ethnicity				
White	72.5	624	72.7	627
People of Color	27.5	237	27.3	235
Black	7.1	61	5.5	47
Hispanic or Latino *	9.3	80	9.9	85
Asian	2.3	38	2.6	22
American Indian	1.5	13	2.4	21
Hawaiian/Pacific Islander	0.5	4	0.3	3
Biracial or Multiracial	4.4	38	4.5	39
Another race/ethnicity	2.4	21	2.1	18
Political Affiliation				
Democrat	65.6	565	62.3	537
Republican	6.3	54	6.1	53
Independent	28.1	242	31.6	272
Relationship Status				
Single	51.6	444	57.3	494
Divorced	2.3	20	1.9	16
Widowed	2.0	17	1.6	14
In a relationship	23.8	205	25.3	218
Married	20.3	175	17.4	150
	*M* (*SD*)	Range	*M* (*SD*)	Range
Age	41.87 (13.36)	19–79	41.35 (13.36)	19–74
Socioeconomic status	2.83 (0.94)	1–5	2.88 (0.92)	1–5
Masculinity	3.61 (0.77)	1–5	3.65 (0.75)	1–5
Perceived weight	3.76 (0.99)	1–9	3.69 (1.03)	1–9

* Participants who identified as Hispanic/Latino were categorized as such (regardless of race).

**Table 2:** Factor Loadings, Means, Standard Deviations, and Cronbach’s Alpha (*n* = 862).

Six-Factor Solution for the G-SISI							
Items	Age Stigma *M* = 2.20 *SD* = 0.94 α = 0.80	Socioeconomic Stigma *M* = 2.31 *SD* = 0.92 α = 0.78	Gay Non-Conformity Stigma *M* = 2.70 *SD* = 0.91 α = 0.60	Racial Stigma *M* = 2.10 *SD* = 0.96 α = 0.81	Gender Expression Stigma *M* = 2.05 *SD* = 0.92 α = 0.85	Body Stigma *M* = 2.67 *SD* = 1.01 α = 0.77	Uniqueness
1. I have been excluded from being asked to participate in activities based on my age.	0.80						0.10
2. I have been treated with less dignity and respect because of my age.	0.76						0.12
3. I have been criticized for being at a gay establishment or gathering because of my age.	0.71						0.15
4. I have been mistreated because of my perceived socioeconomic status.		0.81					0.10
5. I have been left out of group gatherings based on my perceived socioeconomic status.		0.79					0.11
6. I have been judged on my employment status and perceived level of income.		0.63					0.20
7. I have been teased for having interests and hobbies that are not typical of other gay men.			0.75				0.13
8. I have been told I do not accurately represent the cultural norms and stereotypes of a gay man.			0.60				0.22
9. I have received criticism for my political beliefs or party affiliations.			0.46				0.32
10. I have been treated with less respect based on my race/ethnicity at a gay establishment and/or gathering.				0.81			0.10
11. I have heard derogatory jokes and comments about people of my race/ethnicity at a gay bar and/or gathering.				0.74			0.13
12. I have been accused of false stereotypes based on my ethnicity or race.				0.74			0.13
13. I have been desired by someone of another race or ethnicity as a means to fulfill a fetish.				0.58			0.23
14. I have been told I need to behave more masculine or feminine.					0.86		0.07
15. I have been told I should be more or less “straight-acting.”					0.80		0.10
16. I have been called derogatory names and harassed for my gender expression (too feminine or masculine).					0.74		0.13
17. I have been teased for the way I express my gender at gay establishments and/or gatherings.					0.65		0.19
18. I have been told I should gain or lose weight.						0.74	0.13
19. I have been criticized for my body’s level of muscularity (too little or too much muscle).						0.73	0.14
20. I have been physically touched while being told I should change something about my appearance or body size.						0.72	0.15

**Table 3:** Bivariate Correlations Between Subscales (*n* = 862).

G-SISI Subscale	1	2	3	4	5	6
1. Age Stigma	--					
2. Socioeconomic Stigma	0.57 *	--				
3. Gay Non-Conformity Stigma	0.42 *	0.44 *	--			
4. Racial Stigma	0.32 *	0.41 *	0.38 *	--		
5. Gender Expression Stigma	0.41 *	0.48 *	0.41 *	0.45 *	--	
6. Body Stigma	0.43 *	0.51 *	0.42 *	0.40 *	0.61 *	--

* *p* < 0.001.

## Data Availability

The data analyzed during the current study are not publicly available but are available from the corresponding author upon reasonable request.

## References

[1] Porges S.W. (2022). Polyvagal theory: A science of safety. Front. Integr. Neurosci..

[2] Cacioppo J.T., Cacioppo S. (2014). Social relationships and health: The toxic effects of perceived social isolation. Soc. Personal. Psychol. Compass.

[3] Wilson L.C., Liss M. (2022). Safety and belonging as explanations for mental health disparities among sexual minority college students. Psychol. Sex. Orientat. Gend. Divers..

[4] Diamond L.M., Alley J. (2022). Rethinking minority stress: A social safety perspective on the health effects of stigma in sexually-diverse and gender-diverse populations. Neurosci. Biobehav. Rev..

[5] Earnshaw V.A., Watson R.J., Eaton L.A., Brousseau N.M., Laurenceau J.P., Fox A.B. (2022). Integrating time into stigma and health research. Nat. Rev. Psychol..

[6] Ryan C., Huebner D., Diaz R.M., Sanchez J. (2009). Family rejection as a predictor of negative health outcomes in white and Latino lesbian, gay, and bisexual young adults. Pediatrics.

[7] Katz-Wise S.L., Rosario M., Tsappis M. (2016). Lesbian, gay, bisexual, and transgender youth and family acceptance. Pediatr. Clin. North Am..

[8] Evans C.B., Chapman M.V. (2014). Bullied youth: The impact of bullying through lesbian, gay, and bisexual name calling. Am. J. Orthopsychiatry.

[9] Earnshaw V.A., Reisner S.L., Juvonen J., Hatzenbuehler M.L., Perrotti J., Schuster M.A. (2017). LGBTQ Bullying: Translating research to action in pediatrics. Pediatrics.

[10] Seelman K.L., Colón-Diaz M.J.P., LeCroix R.H., Xavier-Brier M., Kattari L. (2017). Transgender noninclusive healthcare and delaying care because of fear: Connections to general health and mental health among transgender adults. Transgend. Health.

[11] Medina-Martínez J., Saus-Ortega C., Sánchez-Lorente M.M., Sosa-Palanca E.M., García-Martínez P., Mármol-López M.I. (2021). Health inequities in LGBT people and nursing interventions to reduce them: A systematic review. Int. J. Environ. Res. Public Health.

[12] Tylka T.L., Annunziato R.A., Burgard D., Daníelsdóttir S., Shuman E., Davis C., Calogero R.M. (2014). The weight-inclusive versus weight-normative approach to health: Evaluating the evidence for prioritizing well-being over weight loss. J. Obes..

[13] Jaspal R. (2022). Social psychological aspects of gay identity development. Curr. Opin. Psychol..

[14] Berg R.C., Munthe-Kaas H.M., Ross M.W. (2016). Internalized homonegativity: A systematic mapping review of empirical research. J. Homosex..

[15] Frost D.M., Fingerhut A.W., Meyer I.H. (2022). Social change and relationship quality among sexual minority individuals: Does minority stress still matter?. J. Marriage Fam..

[16] Van Laar C., Meeussen L., Veldman J., Van Grootel S., Sterk N., Jacobs C. (2019). Coping with stigma in the workplace: Understanding the role of threat regulation, supportive factors, and potential hidden costs. Front. Psychol..

[17] Flentje A., Clark K.D., Cicero E., Capriotti M.R., Lubensky M.E., Sauceda J., Neilands T.B., Lunn M.R., Obedin-Maliver J. (2022). Minority stress, structural stigma, and physical health among sexual and gender minority individuals: Examining the relative strength of the relationships. Ann. Behav. Med. A Publ. Soc. Behav. Med..

[18] Meyer I.H., Russell S.T., Hammack P.L., Frost D.M., Wilson B.D.M. (2021). Minority stress, distress, and suicide attempts in three cohorts of sexual minority adults: A U.S. probability sample. PLoS ONE.

[19] Meyer I.H. (2003). Prejudice, social stress, and mental health in lesbian, gay, and bisexual populations: Conceptual issues and research evidence. Psychol. Bull..

[20] Meyer I.H. (2015). Resilience in the study of minority stress and health of sexual and gender minorities. Psychol. Sex. Orientat. Gend. Divers..

[21] Hatzenbuehler M.L. (2009). How does sexual minority stigma “get under the skin”? A psychological mediation framework. Psychol. Bull..

[22] Muldoon O.T., Haslam S.A., Haslam C., Cruwys T., Kearns M., Jetten J. (2019). The social psychology of responses to trauma: Social identity pathways associated with divergent traumatic responses. Eur. Rev. Soc. Psychol..

[23] Burson E., Godfrey E.B. (2020). Intraminority solidarity: The role of critical consciousness. Eur. J. Soc. Psychol..

[24] Salfas B., Rendina H.J., Parsons J.T. (2019). What is the role of the community? Examining minority stress processes among gay and bisexual Men. Stigma Health.

[25] Foster-Gimbel O., Doyle D.M., Engeln R. (2020). The Gay Community Involvement Index: An exploratory factor analysis and initial validation of a new measure of gay community involvement. Arch. Sex. Behav..

[26] Toomey R.B., Ryan C., Diaz R.M., Russell S.T. (2018). Coping with sexual orientation-related minority stress. J. Homosex..

[27] Mereish E.H., Poteat V.P. (2015). The conditions under which growth-fostering relationships promote resilience and alleviate psychological distress among sexual minorities: Applications of Relational Cultural Theory. Psychol. Sex. Orientat. Gend. Divers..

[28] Branton S.E., Compton C.A. (2021). There’s no such thing as a gay bar: Co-sexuality and the neoliberal branding of queer spaces. Manag. Commun. Q..

[29] Cargnino M., Lemke R. (2022). Online dating site use to cope with minority stress among gay and bisexual men in Germany: Findings from two survey studies. PsyArXiv.

[30] Zervoulis K., Smith D.S., Reed R., Dinos S. (2020). Use of ‘gay dating apps’ and its relationship with individual well-being and sense of community in men who have sex with men. Psychol. Sex..

[31] McConnell E.A., Janulis P., Phillips G., Truong R., Birkett M. (2018). Multiple minority stress and LGBT community resilience among sexual minority men. Psychol. Sex. Orientat. Gend. Divers..

[32] Kousari-Rad P., McLaren S. (2013). The relationships between sense of belonging to the gay community, body image dissatisfaction, and self-esteem among Australian gay men. J. Homosex..

[33] Foster-Gimbel O., Engeln R. (2016). Fat chance! Experiences and expectations of antifat bias in the gay male community. Psychol. Sex. Orientat. Gend. Divers..

[34] Parmenter J.G., Galliher R.V. (2022). Development and initial validation of the LGBTQ+ Community Resilience and Inequity Scale. Psychol. Sex. Orientat. Gend. Divers..

[35] Pachankis J.E., Clark K.A., Burton C.L., Hughto J., Bränström R., Keene D.E. (2020). Sex, status, competition, and exclusion: Intraminority stress from within the gay community and gay and bisexual men’s mental health. J. Personal. Soc. Psychol..

[36] Convertino A.D., Brady J.P., Albright C.A., Gonzales M., Blashill A.J. (2021). The role of sexual minority stress and community involvement on disordered eating, dysmorphic concerns and appearance- and performance-enhancing drug misuse. Body Image.

[37] Maiolatesi A.J., Satyanarayana S., Bränström R., Pachankis J.E. (2021). Development and validation of two abbreviated intraminority gay community stress scales. Assessment.

[38] Mendoza-Denton R., Page-Gould E., Pietrzak J., Levin S., van Laar C. (2006). Mechanisms for coping with status-based rejection expectations. Stigma and Group Inequality: Social Psychological Perspectives.

[39] Kler S., Shepherd B.F., Renteria R. (2022). Community connectedness as a moderator of the association between intersectional microaggressions and alcohol use among sexual and gender minoritized people of color. Subst. Use Misuse.

[40] Petruzzella A., Feinstein B.A., Davila J., Lavner J.A. (2019). Moderators of the association between community connectedness and internalizing symptoms among gay men. Arch. Sex. Behav..

[41] Harris A.C. (2009). Marginalization by the marginalized: Race, homophobia, heterosexism, and “the problem of the 21st century”. J. Gay Lesbian Soc. Serv..

[42] Wade R.M., Pear M.M. (2022). Online dating and mental health among young sexual minority Black men: Is ethnic identity protective in the face of sexual racism?. Int. J. Environ. Res. Public Health.

[43] Han C.S., Choi K.H. (2018). Very few people say “No Whites”: Gay men of color and the racial politics of desire. Sociol. Spectr..

[44] Ramirez J.L., Galupo M.P. (2019). Multiple minority stress: The role of proximal and distal stress on mental health outcomes among lesbian, gay, and bisexual people of color. J. Gay Lesbian Ment. Health.

[45] Jackson S.D., Mohr J.J., Sarno E.L., Kindahl A.M., Jones I.L. (2022). Intersectional experiences, stigma-related stress, and psychological health among Black LGBQ individuals. J. Consult. Clin. Psychol..

[46] Crenshaw K. (1989). Demarginalizing the intersection of race and sex: A Black Feminist Critique of Antidiscrimination Doctrine, Feminist Theory and Antiracist Politics. Univ. Chic. Leg. Forum.

[47] Crenshaw K. (1991). Mapping the margins: Intersectionality, identity politics, and violence against women of color. Stanf. Law Rev..

[48] Bauer G.R., Scheim A.I. (2019). Methods for analytic intercategorical intersectionality in quantitative research: Discrimination as a mediator of health inequalities. Soc. Sci. Med..

[49] Maki J. (2018). A Quantitative Study of Within-Group Discrimination of Gay Men. Doctoral Dissertation.

[50] Hoy-Ellis C.P., Ator M., Kerr C., Milford J. (2016). Innovative approaches address aging and mental health needs in LGBTQ communities. Generations.

[51] Wight R.G., LeBlanc A.J., Meyer I.H., Harig F.A. (2015). Internalized gay ageism, mattering, and depressive symptoms among midlife and older gay-identified men. Soc. Sci. Med..

[52] Barrett D.C., Pollack L.M. (2005). Whose gay community? Social class, sexual self-expression, and gay community involvement. Sociol. Q..

[53] McGarrity L.A. (2014). Socioeconomic status as context for minority stress and health disparities among lesbian, gay, and bisexual individuals. Psychol. Sex. Orientat. Gend. Divers..

[54] Worthen M.G.F. (2020). “All the gays are liberal?” Sexuality and gender gaps in political perspectives among lesbian, gay, bisexual, mostly heterosexual, and heterosexual college students in the Southern USA. Sex. Res. Soc. Policy.

[55] Hunt C.J., Fasoli F., Carnaghi A., Cadinu M. (2016). Masculine self-presentation and distancing from femininity in gay men: An experimental examination of the role of masculinity threat. Psychol. Men Masc..

[56] Miller B., Behm-Morawitz E. (2016). “Masculine guys only”: The effects of femmephobic mobile dating application profiles on partner selection for men who have sex with men. Comput. Hum. Behav..

[57] Austen E., Greenaway K.H., Griffiths S. (2020). Differences in weight stigma between gay, bisexual, and heterosexual men. Body Image.

[58] Green A.I. (2014). Sexual Fields: Toward a Sociology of Collective Sexual Life.

[59] Boateng G.O., Neilands T.B., Frongillo E.A., Melgar-Quiñonez H.R., Young S.L. (2018). Best practices for developing and validating scales for health, social, and behavioral research: A primer. Front. Public Health.

[60] Roberts L.M., Christens B.D. (2021). Pathways to well-being among lgbt adults: Sociopolitical involvement, family support, outness, and community connectedness with race/ethnicity as a moderator. Am. J. Community Psychol..

[61] George D., Mallery M. (2010). SPSS for Windows Step by Step: A Simple Guide and Reference, 17.0 Update.

[62] Kim H.Y. (2013). Statistical notes for clinical researchers: Assessing normal distribution (2) using skewness and kurtosis. Restor. Dent. Endod..

[63] Worthington R.L., Whittaker T.A. (2006). Scale development research: A content analysis and recommendations for best practices. Couns. Psychol..

[64] Quintana S.M., Maxwell S.E. (1999). Implications of recent developments in structural equation modeling for counseling psychology. Couns. Psychol..

[65] Weston R., Gore P.A. (2006). A brief guide to structural equation modeling. Couns. Psychol..

[66] Muthén L.K., Muthén B.O. (1998–2017). Mplus User’s Guide.

[67] Cohen J. (1988). Statistical Power Analysis for the Behavioral Sciences.

[68] Funder D.C., Ozer D.J. (2019). Evaluating effect size in psychological research: Sense and nonsense. Adv. Methods Pract. Psychol. Sci..

[69] Kaiser H.F. (1974). An index of factorial simplicity. Psychometrika.

[70] Bartlett M.S. (1951). A further note on tests of significance in factor analysis. Br. J. Psychol..

[71] Kyriazos T.A. (2018). Applied psychometrics: Sample size and sample power considerations in factor analysis (EFA, CFA) and SEM in general. Psychology.

[72] Streiner D.L. (2003). Starting at the beginning: An introduction to coefficient alpha and internal consistency. J. Personal. Assess..

[73] Filice E., Raffoul A., Meyer S.B., Neiterman E. (2019). The influence of Grindr, a geosocial networking application, on body image in gay, bisexual and other men who have sex with men: An exploratory study. Body Image.

[74] Else-Quest N.M., Hyde J.S. (2016). Intersectionality in quantitative psychological research: II. Methods and techniques. Psychol. Women Q..

[75] Mahon C.P., Pachankis J.E., Kiernan G., Gallagher P. (2021). Risk and protective factors for social anxiety among sexual minority individuals. Arch. Sex. Behav..

[76] Burton C.L., Clark K.A., Pachankis J.E. (2020). Risk from within: Intraminority gay community stress and sexual risk-taking among sexual minority men. Ann. Behav. Med. A Publ. Soc. Behav. Med..

[77] Balsam K.F., Molina Y., Beadnell B., Simoni J., Walters K. (2011). Measuring multiple minority stress: The LGBT People of Color Microaggressions Scale. Cult. Divers. Ethn. Minor. Psychol..

[78] Zelaya D.G., DeBlaere C., Velez B.L. (2021). Psychometric validation and extension of the LGBT people of Color Microaggressions Scale with a sample of sexual minority BIPOC college students. Psychol. Sex. Orientat. Gend. Divers..

[79] Huynh K.D., Bricker N.L., Lee D.L., Balsam K.F. (2022). Development and validation of the LGBTQ+ POC Microaggressions Scale—Brief (LGBTQ+ PCMS-B). Stigma Health.

[80] Friedland B.A., Sprague L., Nyblade L., Baral S.D., Pulerwitz J., Gottert A., Amanyeiwe U., Cheng A., Mallouris C., Anam F. (2018). Measuring intersecting stigma among key populations living with HIV: Implementing the people living with HIV Stigma Index 2.0. J. Int. AIDS Soc..

[81] Rodríguez-Roldán V.M. (2020). The Intersection between Disability and LGBT Discrimination and Marginalization. Am. Univ. J. Gend. Soc. Policy Law.

[82] Levine E.C., Herbenick D., Martinez O., Fu T.C., Dodge B. (2018). Open relationships, nonconsensual nonmonogamy, and monogamy among U.S. adults: Findings from the 2012 National Survey of Sexual Health and Behavior. Arch. Sex. Behav..

[83] Ponterotto J.G., Ruckdeschel D.E. (2007). An overview of coefficient alpha and a reliability matrix for estimating adequacy of internal consistency coefficients with psychological research measures. Percept. Mot. Ski..

[84] Lagos D., Compton D. (2021). Evaluating the use of a two-step gender identity measure in the 2018 General Social Survey. Demography.

[85] Beischel W.J., Schudson Z.C., Hoskin R.A., van Anders S.M. (2022). The gender/sex 3×3: Measuring and categorizing gender/sex beyond binaries. Psychol. Sex. Orientat. Gend. Divers..

[86] Soulliard Z.A., Layland E.K., Smith J.C., Kipke M.D., Bray B.C. (2022). Body image concerns, correlates, and community connection among Black and Latinx sexual minority cisgender men and transgender/gender nonconforming young adults. LGBT Health.

[87] Maki J.L. (2017). Gay subculture Identification: Training Counselors to Work with Gay Men. Vistas.

[88] Moskowitz D.A., Turrubiates J., Lozano H., Hajek C. (2013). Physical, behavioral, and psychological traits of gay men identifying as bears. Arch. Sex. Behav..

[89] Prestage G., Brown G., De Wit J., Bavinton B., Fairley C., Maycock B., Batrouney C., Keen P., Down I., Hammoud M. (2015). Understanding gay community subcultures: Implications for HIV prevention. AIDS Behav..

[90] Antebi-Gruszka N., Friedman A.A., Balsam K.F. (2022). Multiple forms of discrimination, mental distress, and well-being among lesbian, gay, bisexual, and queer individuals: The role of brooding. J. Gay Lesbian Ment. Health.

[91] Shepherd B., Kelly L.M., Brochu P.M., Wolff J., Swenson L. (2023). An examination of theory-based suicidal ideation risk factors in college students with multiple marginalized identities. Am. J. Orthopsychiatry.

[92] Bostwick W.B., Boyd C.J., Hughes T.L., West B.T., McCabe S.E. (2014). Discrimination and mental health among lesbian, gay, and bisexual adults in the United States. Am. J. Orthopsychiatry.

[93] Convertino A.D., Elbe C.I., Mendoza R.R., Calzo J.P., Brown T.A., Siegel J.A., Jun H.J., Corliss H.L., Blashill A.J. (2022). Internalization of muscularity and thinness ideals: Associations with body dissatisfaction, eating disorder symptoms, and muscle dysmorphic symptoms in at risk sexual minority men. Int. J. Eat. Disord..

[94] Shepherd B.F., Brochu P.M., Rodriguez-Seijas C. (2022). A critical examination of disparities in eating disorder symptoms by sexual orientation among US adults in the NESARC-III. Int. J. Eat. Disord..

[95] Rodriguez-Seijas C., Eaton N.R., Pachankis J.E. (2019). Prevalence of psychiatric disorders at the intersection of race and sexual orientation: Results from the National Epidemiologic Survey of Alcohol and Related Conditions-III. J. Consult. Clin. Psychol..

[96] Choi S.K., Meyer I.H. (2016). LGBT Aging: A Review of Research Findings, Needs, and Policy Implications.

[97] Shepherd B.F., Chang C.M., Dyar C., Brochu P.M., Selby E.A., Feinstein B.A. (2022). Out of the closet, but not out of the woods: The longitudinal associations between identity disclosure, discrimination, and non-suicidal self-injury among sexual minoritized young adults. Psychol. Sex. Orientat. Gend. Divers..

[98] Kelly L.M., Shepherd B.F., Becker S.J. (2021). Elevated risk of substance use disorder and suicidal ideation among Black and Hispanic lesbian, gay, and bisexual adults. Drug Alcohol Depend..

[99] Dziengel L. (2015). A be/coming-out model: Assessing factors of resilience and ambiguity. J. Gay Lesbian Soc. Serv..

[100] Kidd S.A., Howison M., Pilling M., Ross L.E., McKenzie K. (2016). Severe mental illness in LGBT populations: A scoping review. Psychiatr. Serv..

[101] Bishop J., Crisp D., Scholz B. (2022). The real and ideal experiences of what culturally competent counselling or psychotherapy service provision means to lesbian, gay and bisexual people. Couns. Psychother. Res..

[102] Adames H.Y., Chavez-Dueñas N.Y., Sharma S., La Roche M.J. (2018). Intersectionality in psychotherapy: The experiences of an AfroLatinx queer immigrant. Psychotherapy.

[103] Alvarez J.C., Waitz-Kudla S., Brydon C., Crosby E., Witte T.K. (2022). Culturally responsive scalable mental health interventions: A call to action. Transl. Issues Psychol. Sci..

[104] Pachankis J.E., Soulliard Z.A., Morris F., van Dyk I.S. (2022). A model for adapting evidence-based interventions to be LGBQ-affirmative: Putting minority stress principles and case conceptualization into clinical research and practice. Cogn. Behav. Pract..

[105] Quinn K.G., Dickson-Gomez J., Pearson B., Marion E., Amikrhanian Y., Kelly J.A. (2022). Intersectional resilience among Black gay, bisexual, and other men who have sex with men, Wisconsin and Ohio, 2019. Am. J. Public Health.

[106] Bogart L.M., Galvan F.H., Leija J., MacCarthy S., Klein D.J., Pantalone D.W. (2020). A pilot Cognitive Behavior Therapy Group intervention to address coping with discrimination among HIV-positive Latino immigrant sexual minority men. Ann. LGBTQ Public Popul. Health.

[107] Gonzales G., Gavulic K.A. (2020). The Equality Act is needed to advance health equity for Lesbian, Gay, Bisexual, and Transgender populations. Am. J. Public Health.

[108] Cech E.A., Rothwell W.R. (2020). LGBT workplace inequality in the federal workforce: Intersectional processes, organizational contexts, and turnover considerations. ILR Rev..

[109] Zelaya D.G., Guy A.A., Surace A., Mastroleo N.R., Pantalone D.W., Monti P.M., Mayer K.H., Kahler C.W. (2022). Modeling the impact of race, socioeconomic status, discrimination and cognitive appraisal on mental health concerns among heavy drinking HIV+ cisgender MSM. AIDS Behav..

[110] Bränström R., Pachankis J. (2022). Structural stigma and 7-year improvement in life satisfaction: A repeated cross-sectional study of sexual minority individuals across 28 countries. Eur. J. Public Health.

